# Ultrabroadband Absorption Enhancement via Hybridization of Localized and Propagating Surface Plasmons

**DOI:** 10.3390/nano10091625

**Published:** 2020-08-19

**Authors:** Tian Sang, Honglong Qi, Xun Wang, Xin Yin, Guoqing Li, Xinshang Niu, Bin Ma, Hongfei Jiao

**Affiliations:** 1 Department of Photoelectric Information Science and Engineering, School of Science, Jiangnan University, Wuxi 214122, China; 18860478830@163.com (H.Q.); wangxun1012gg@163.com (X.W.); xinyin0202@163.com (X.Y.); 18351037802@163.com (G.L.); 2 Key Laboratory of Advanced Micro-Structured Materials MOE Institute of Precision Optical Engineering, School of Physics Science and Engineering, Tongji University, Shanghai 200092, China; 1631921@tongji.edu.cn (X.N.); mabin@tongji.edu.cn (B.M.)

**Keywords:** metamaterial absorbers, ultrabroadband absorption, localized surface plasmon, propagating surface plasmon

## Abstract

Broadband metamaterial absorbers (MAs) are critical for applications of photonic and optoelectronic devices. Despite long-standing efforts on broadband MAs, it has been challenging to achieve ultrabroadband absorption with high absorptivity and omnidirectional characteristics within a comparatively simple and low-cost architecture. Here we design, fabricate, and characterize a novel compact Cr-based MA to achieve ultrabroadband absorption in the visible to near-infrared wavelength region. The Cr-based MA consists of Cr nanorods and Cr substrate sandwiched by three pairs of SiO_2_/Cr stacks. Both simulated and experimental results show that an average absorption over 93.7% can be achieved in the range of 400–1000 nm. Specifically, the ultrabroadband features result from the co-excitations of localized surface plasmon (LSP) and propagating surface plasmon (PSP) and their synergistic absorption effects, where absorption in the shorter and longer wavelengths are mainly contributed bythe LSP and PSP modes, respectively. The Cr-based MA is very robust to variations of the geometrical parameters, and angle-and polarization-insensitive absorption can be operated well over a large range of anglesunder both transverse magnetic(TM)- and transverse electric (TE)-polarized light illumination.

## 1. Introduction

Light absorption enhancements in metamaterials have received intensive attention due to their diverse performances in a series of applications such as thermal emitters [1,2], sensors [3,4,5], photodetectors [6,7], photovoltaics [8,9], and optical imaging devices [10,11,12]. In general, the typical design scheme for a metamaterial absorber (MA) consists of sandwiched metal-insulator-metal (MIM) layers with the patterned top surface [13]. In these structures, the electric dipole resonance can be induced by the metallic patterns and the magnetic dipole resonance can be excited by the anti-parallel currents between the interfaces of the metal and insulator layers. By using different patterned surfaces such as strip [14], square [15], hole [16], and disk [17], perfect absorption can be achieved as the impedance of the structure equals that of the incident medium at a specific frequency. Furthermore, by using more sophisticated metallic patterns such as crossed trapezoid [18], nanostars [19], and square ring [20], the absorption bandwidth can be improved to cover the whole visible region. However, the absorption bandwidth of the MIM-based MA is relatively narrow due to the resonance effect of the magnetic or electric dipole.

To extend the bandwidth, multi-sized resonators with patterns such as strips [21,22], patches [23,24], ellipses [25], and crosses [26] can be integrated together to form a supercell, and the absorption bandwidth of the multi-sized MA can be effectively improved due to the blending of multiple resonant frequencies. Unfortunately, the achievable bandwidth is limited because the number of possible integrated resonators in a supercell is restricted. Moreover, with the increase of the number of the resonators, the effective number densityof each resonator decreases, resulting in deteriorationof the overall absorption performances [27].

In recent years, hyperbolic metamaterials (HMMs) have been deemed a valuable class of optical metamaterials to enhance light-matter interaction due to the anisotropic optical response. Such anisotropic behavior is accompanied by an isofrequency surface in the shape of a hyperboloid, which supports propagating high wavevector modes and demonstrates an enhanced photonic density of states [28,29,30].Moreover, HMMs have been demonstrated to enable a tailored control of light extinction, scattering, and absorption processes [31,32,33], to achieve resonant gain singularities [34] and enhanced spontaneous emission rates of molecules [35,36,37].Recently, a mechanismhas been proposed to achieve high absorption (experimentally >90%, theoretically approaching 99%) in the near-infrared region [38], where hybrid localizedpropagating modes are responsible for the excitation of highly absorptive Bloch Plasmon polariton modes supported by the multilayered HMM structure. Similarly, near-perfect absorption is also achievable by exploiting the direct excitation of localized and/or propagating modes within the HMM structures [39,40,41]. In particular, the taped metal/dielectric stack-based HMMs, such as sawtooths [42,43,44], nano-cones [45,46,47], and pyramids [48,49], showing the hyperbolic dispersion diagram, enable broadband light absorption enhancement due to the collective excitation of slow-light waveguide modes, i.e., the trapped rainbow effect [50,51]. Although the absorption performances of the taped metal/dielectric stack-based HMMs are striking, their fabrication procedure is quite challenging because graded width of metal/dielectric patterns as well as a large number of thin-film stacks are required. Noble metals are used widely in most MAs due to their excellent performance supporting surface plasmon polaritons at optical frequencies, which leads to expensive cost in the fabrication process. From the point of view of application, despite the elaborate study of MAs, further performance improvementsin absorption efficiency, bandwidth, and most importantly omnidirectional features are highly desired.

Herein, we propose a novel architecture of Cr-based MA with subwavelength thickness to achieve ultrabroadband, angle-and polarization-insensitive absorption in the visible and near-infrared regions. The Cr-based MA consists of Cr nanorods and Cr substrate sandwiched by three pairs of SiO_2_/Cr stacks. Both simulated and experimental results indicate that an average absorption over 93.7% can be achieved at wavelengths ranging from 400 to 1000 nm. The physical basis for the ultrabroadband absorption is the hybridization of localized and propagating surface plasmon modes, where absorption enhancement in the shorter and longer wavelengths are mainly contributed from the localized surface plasmon (LSP) and propagating surface plasmon (PSP) modes, respectively. Moreover, the absorption performances of the Cr-based MA are insensitive to variations of the structural parameters, and the broadband features can be maintained even at the incident angle of 60° for bothtransverse magnetic(TM) and transverse electric (TE) polarizations. All these advantageous optical properties make our proposed structure a good candidate to improve various important applications such as photovoltaic devices, remote sensing and photodetectors.

## 2. Design and Characterization of the Proposed MA

**Figure 1:**
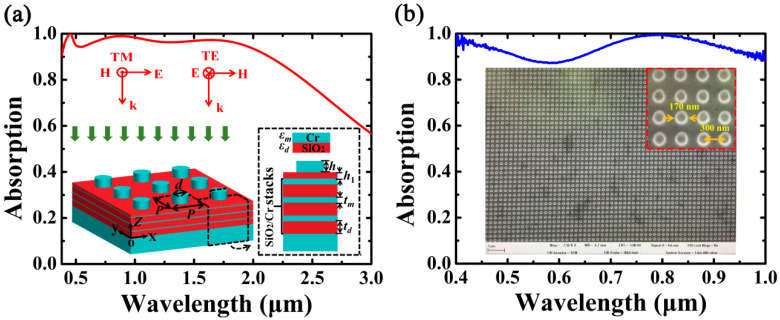
(**a**) A schematic diagram of the Cr-basedmetamaterial absorber(MA) and its simulated absorption in the designed wavelength region. The parameters are: *P* = 300 nm, *d* = 165 nm, *h* = 100 nm, *h*_1_ = 28 nm, *t_m_* = 6 nm, and *t_d_* = 85 nm. (**b**) The measured absorption and the SEM image of the fabricated MA with a zoomed SEM image in the inset.

Figure 1a shows the schematic diagram of the proposed MA and its absorption response under normal illumination of the transverse magnetic (TM) or transverse electric (TE) plane wave. The inset is the side view of the Cr-based MA, and the structural parameters of the unit cell are labeled in the figure caption. The Cr-based MA consists of Cr nanorods and Cr substrate sandwiched by three pairs of SiO_2_/Cr stacks, and the Cr nanorods and SiO_2_/Cr stacks are separated by a SiO_2_ buffer layer with the thickness *h*_1_. The period of the unit cell is *P*, the height and diameter of the Cr nanorods are *h* and *d*, respectively; *t_m_* and *t_d_* are the thickness of Cr and SiO_2_ of the film stacks, respectively. The refractive index of SiO_2_ is 1.47, and the complex refractive indices of the Cr film are obtained fromRakic et al. [52].

In simulations, a finite-difference time-domain (FDTD) approach is adopted to calculate the absorption of the Cr-based MA using the commercial software Lumerical [53]. At normal incidence, periodic boundary conditions are used in the *x* and *y* directions with Bloch/periodic plane wave type, and a perfectly matched layer is used in the *z* direction. However, Bloch boundary conditions need to be chosen in the *x* and *y* directions with BFAST plane wave type at oblique incidence. The grid sizes in the *x*, *y* and *z* directions are 1.5 nm × 1.5 nm × 1.5 nm, respectively.The absorption of the total structure can be reduced as A(*λ*) = 1 − R(*λ*) because there is no transmission for the optically thick Cr substrate, where R(λ) is the reflection of the structure. In addition, the absorption of different parts of the Cr-based structure can be calculated by integrating power dissipation in the desired part of the unit cell [15,54]:

(1) Pabs=12ε0ωIm(ε)∫V|E|2dV

where *ε_0_* is the vacuum permittivity, *ω* is the angular frequency, *ε* is the relative dielectric permittivity. and Im(*ε*) is its imaginary part; |*E*| denotes the electric-field amplitude.

In addition, to compare the absorption bandwidth of the proposed MA with other types of MAs, we define the relative absorption bandwidth (RAB) as:

(2) $$\mathrm{RAB}=2(\lambda_{L}-\lambda_{S})/(\lambda_{L}+\lambda_{S})$$

where *λ_L_* and *λ_S_* are the larger and smaller limits of wavelength with the absorptivity greater than 90%, and the average absorption within the operating wavelength region can be calculated as $A_{\mathrm{av}}=\frac{1}{\lambda_{L}-\lambda_{S}}\int_{\lambda_{S}}^{\lambda_{L}}A(\lambda )d\lambda$.

In Figure 1a, the absorption response of the Cr-based MA is calculated as A = (A_TM_ + A_TE_)/2, where A_TM_ and A_TE_ are the absorption of the TM and TE polarizations, respectively. As shown in Figure 1a, ultrabroadband absorption can be realized at wavelengths ranging from 380.0 to 2137.5 nm with absorptivity higher than 90% and average absorption of *A*_av_ = 96.4%. The RAB of the Cr-based MA is 139.6%, which is larger than many types of MAs such as MIM-based MAs [13,14,15,16,17,18,19,20], multi-sized MAs [21,22,23,24,25,26], and HMM-based MAs [42,43,44,45,46]. In addition, although only three metal/dielectric stacks are required, the RAB of the Cr-based MA is also comparable with those of the MAs such as multilayered-based nanocones [47] and pyramids [48,49]. Therefore, the proposed MA possesses the advantages of combing excellent absorption performance with comparatively simple and low-cost configuration.

The SEM image of the fabricated MA and the experimental absorption spectrum are depicted in Figure 1b. The Cr-based MA is achieved by combining the thin-films deposition with nanorod array fabrication. In the process of Cr/SiO_2_ thin-films deposition, we used the e-beam evaporation coater (OTFC-1300, OPTORUN Co. Ltd., Saitama, Japan) whichwas equipped with two JEOL electron guns and an ion-source. The Cr substrate, SiO_2_/Cr stacks, and SiO_2_ buffer layer were deposited alternatively by OTFC-1300 on K9 glasses with a diameter of 25 mm. The Cr films were deposited by e-beam evaporation with a deposition rate of 0.7 nm/s and pressure of 4.3 × 10^−3^ Pa; the SiO_2_ films were deposited by ion-assisted deposition with a deposition rate of 0.8 nm/s and pressure of 7.3 × 10^−3^ Pa. The Cr nanorod arrays were fabricated as follows. Firstly, a 380 nm thick e-beam resist (Zep 520) was spin-coated with 4000 rpm on the Cr/SiO_2_ thin-films and baked on a hotplate at 180 °C for 180 s. Then, the resist layer with an area of 180 × 180 μm was exposed by e-beam writing (JEOL 6300 FS; JEOL Ltd., Akishima, Japan) at 100 kV with a 7 nm beam size. After e-beam exposure, the development process was carried out to realize nanohole arrays using amyl acetate for 70 s, and then isopropyl alcohol (IPA) rinse for 60 s. Finally, a 100 nm thick Cr film was deposited on the nanohole array via e-beam evaporation, andthe remained e-beam resist was removed by N-Methyl-2-Pyrrolidinone (NMP) and DI water to obtain the proposed MA.

In Figure 1b, the reflection spectra of the sample are measured by an angle-resolved microspectroscope (AR-ARM; Ideaoptics Instruments Co. Ltd., China). A 100 W halogen lamp is used to produce a broadband illumination. Measurement is set to an area about 80 × 80 μm using a 100 × microscope objective lens with a numerical aperture of 0.9. Reflection spectra R(*λ*) were normalized to a silver mirror with 96% reflectivity in the designed wavelength region, and the measured absorption of the sample can be obtained as A(*λ*) = 1 − R(*λ*). Herein, the wavelength range is confined within 400–1000 nm due to the limitation of detectability of the silicon-based photodetector of our measured system. As can be seen in Figure 1b, the measured results agree well with the simulations in structural profile as well as absorption performance, and an average absorption of 93.7% can be achieved within the wavelength region of 400–1000 nm. However, there are some mismatches between the simulation and experimental results; for example, the two absorption peaks in the shorter and longer wavelengths in simulation are 451.2 nm and 884.5 nm, respectively; while in experiment they are blue-shifted to 418.8 nm and 802.8 nm, respectively. The discrepancies may result from fabrication errors, such as surface/edge roughness of the Cr nanorods, and deviations in the realized dimensions. In addition, the inconsistent permittivity of Cr between the simulation and experiment is also responsible for the mismatches between the simulation and experimental results.

## 3. Physical Basis for Ultrabroadband Absorption Enhancement

**Figure 2:**
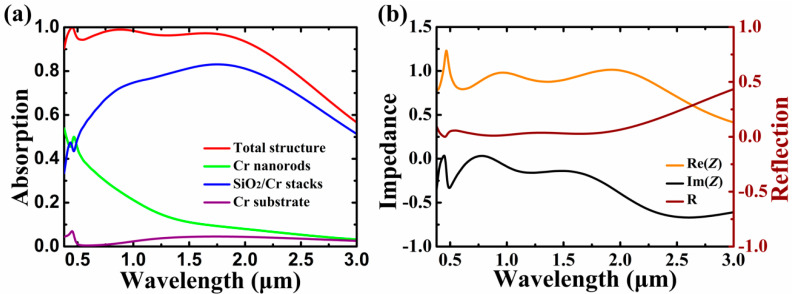
(**a**) Absorption of the Cr-based MA and its absorption distributions in different parts of the structure. (**b**) Impedance and reflection curves of the Cr-based MA. Other parameters are kept the same as Figure 1a.

To better understand the physical basis for ultrabroadband absorption of the proposed MA, the absorption of the total structure and its distributions in different parts of the structure are shown in Figure 2a. As can be seen in Figure 2a, the absorption of the total structure is mainly contributed bythe SiO_2_/Cr stacks, and the absorption of the Cr substrate is comparably low in the whole wavelength region. However, absorption contributed bythe Cr nanorods plays an important role inrealizing high-efficiency absorption of the total structure; in particular, a large amount of absorption is resulted from the Cr nanorods in the short wavelength region. To corroborate the correlation between the absorption performances and the related electromagnetic parameters of the Cr-based metasurface, here we investigate the input impedances of the total structure by using the impedance theory. Based on the impedance theory [55,56], the impedance *Z* of the Cr-based MA can be obtained by the extraction of the scattering parameters as follows:

(3) $$Z=\pm \sqrt{\frac{{\left(1+S_{11}\right)}^{2}-S_{21}^{2}}{{\left(1-S_{11}\right)}^{2}-S_{21}^{2}}}$$

(4) $$S_{11}=S_{22}=\frac{i(1-Z^{2})\sin(nkD)}{2Z}$$

(5) $$S_{21}=S_{12}=\frac{4}{4\cos(nkd)-i(2Z+1)\sin(nkd)}$$

where *S*_11_, *S*_22_, *S*_21_, *S*_12_ are *S* parameters; *k*, *n*, and *D* are the wave vector, the effective refractive index, and thickness of the structure, respectively. The reflection of the Cr-based MA can be calculated as R = [(*Z* − *Z*_0_)/(*Z* + *Z*_0_)]^2^, where *Z* and *Z*_0_ are the normalized impedance of the structure and free space, respectively. In simulation, S parameters of the Cr-based metasurface are complex amplitude reflection and transmission coefficients, which can be obtained by using the S parameters analysis group of Lumerical. As can be seen in Figure 2b, the real part of *Z* tends to 1 and its imaginary part approaches 0 in the range of 0.38–2.14 μm, leading to the ultrabroadband antireflection effect with R ≈ 0 in the wavelength region of interest.

**Figure 3:**
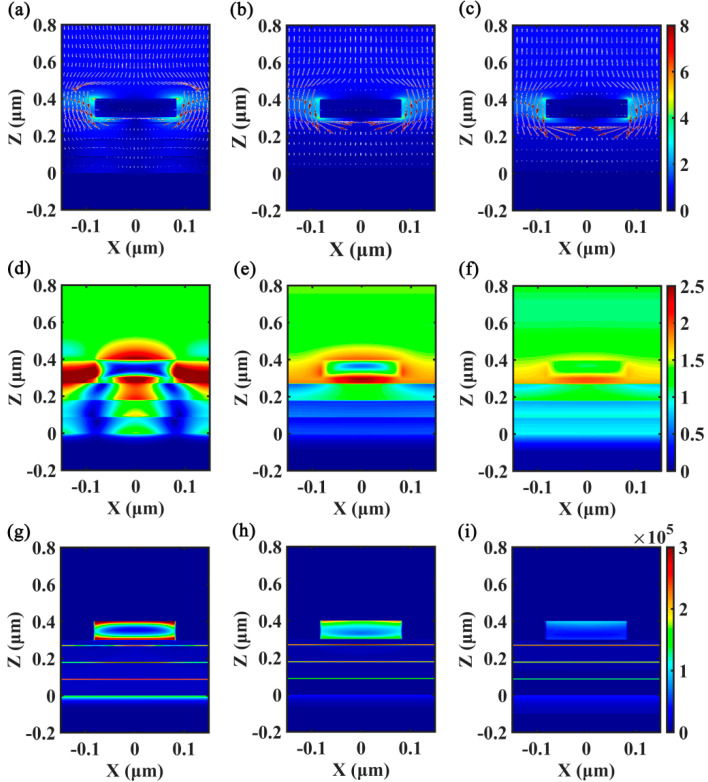
Distributions of electromagnetic field, Poynting vector, and current density associated with the absorption peaks of the Cr-based MA. Other parameters are the same as Figure 1a. (**a**–**c**) are the near field distributions of |*E*| and Poynting vector at 451.2 nm, 884.5 nm, and 1642.3 nm, respectively. The red arrow indicates the direction of Poynting vector. (**d**–**f**) are the near field distributions of |*H*| at 451.2 nm, 884.5 nm, and 1642.3 nm, respectively. (**g**–**i**) are the distributions of current density at 451.2 nm, 884.5 nm, and 1642.3 nm, respectively.

To gain further insight into the ultrabroadband absorption enhancement of the Cr-based MA, distributions of electromagnetic field, Poynting vector, and current density associated with the absorption peaks are shown in Figure 3. As can be seen in Figure 3a–c, the normalized amplitudes of the electric field of the absorption peaks are distinctly enhanced, and four hotspots occur at both the top and bottom edges of the Cr nanorods, indicating the excitation of LSP, which has a dipolar feature [57,58]. Moreover, the Poynting vector distribution shows that the energy of the incident light primarily flows and dissipates around the Cr nanorods, and more light will penetrate into the SiO_2_/Cr stacks as resonance wavelength is increased. In Figure 3d, the normalized magnetic field distribution shows that the excitation of PSP is evident in the region among the neighboring Cr nanorods, while a large amount of PSP simultaneously propagates into the SiO_2_/Cr stacks and Cr substrate via the buffer layer as well. Note that the magnetic field distribution in Figure 3d is more localized in the SiO_2_/Cr stacks by comparison with that shownin Figure 3e,f due to its high quality resonance feature. In Figure 3e,f, it can be seen that the ratio of PSP that propagates into the SiO_2_/Cr stacks is increased for the longer wavelength, which exhibits a similar trend tothat of the Poynting vector distributions in Figure 3b,c. Therefore, the co-excitations of LSP and PSP and their synergistic absorption effects strongly improve the interaction between light and Cr material, resulting in ultrabroadband absorption of the Cr-based MA with subwavelength thickness. Figure 3g–i shows that the current densities of the absorption peaks are simultaneously enhanced in the Cr nanorods, Cr stacks, and Cr substrate, and the ratio of the current density in the Cr stacks is increased for the longer wavelength, indicating that the absorption contributed bythe SiO_2_/Cr stacks will be increased with the increase of absorption wavelength, a result which isin line with the results in Figure 2a.

## 4. Evaluation of Absorption Performances of the Proposed MA

**Figure 4:**
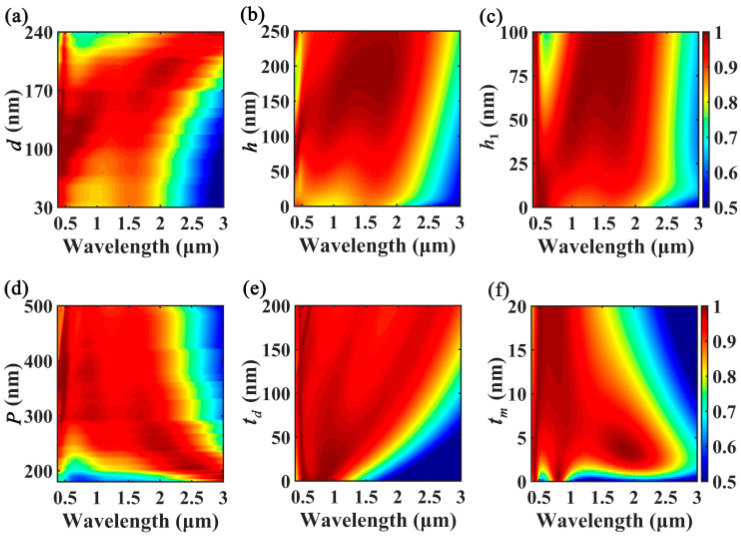
Absorption 2D map of the Cr-based MA as functions of (**a**) diameter of the Cr nanorods *d*, (**b**) height of the Cr nanorods *h*, (**c**) thickness of the buffer layer *h*_1_, (**d**) period *P*, (**e**) thickness of SiO_2_ film *t_d_*, and (**f**) thickness of Cr film *t_m_*. Other parameters are kept the same as Figure 1a.

To evaluate the robustness of absorption performances of the proposed MA, we first investigate the influences of the geometrical parameters (*d*, *h*, *h*_1_, *P*, *t_d_*, *t_m_*) on the absorption responses of the Cr-based MA. As can be seen in Figure 4a, the Cr-based MA shows broadband absorption features as *d* varied in the range of 75–195 nm, and good absorption performances that combine the merits of both broadband absorption and high absorption efficiency can be realized around *d* = 165 nm. Figure 4b shows that ultrabroadband absorption can be realized as the height of the Cr nanorods is large enough (*h* > 70 nm), while the absorption performances will be degraded if *h* is too small, due to the coupling of the surface plasmons between the top and bottom surfaces of the Cr nanorod. In Figure 4c, it can be seen that the absorption performance of the Cr-based MA is not good without the buffer layer (*h*_1_ = 0); however, the absorption curves deteriorate and the absorption band issplit as *h*_1_ > 50 nm. By properly selecting the thickness of the buffer layer such as *h*_1_ = 28 nm, the absorption bandwidth can be effectively improved due to electromagnetic coupling between the Cr nanorods and SiO_2_/Cr stacks. As Figure 4d shows, broadband absorption enhancement can be maintained even if period is significantly varied. However, in the deep-subwavelength region (*P* < 200 nm), the absorption is reduced significantly due to the increase inreflection of the structure. In Figure 4e, it can be seen that the absorption bandwidth can be enlarged by increasing *t_d_*; however, the absorption performances possessing ultrabroadband and high absorption efficiency will be best near *t_d_* = 85 nm. In Figure 4f, it can be seen that the absorption bandwidth can be significantly improved around *t_m_* = 5 nm. Note that broadband absorption will be reduced totwo absorption bands as *t_m_* = 0; the absorption band near *λ* = 0.39 μm is associated with the LSP, while the absorption band near *λ* = 0.80 μmarises from the excitation of gap plasmon of the MIM-based structures [59,60].

**Figure 5:**
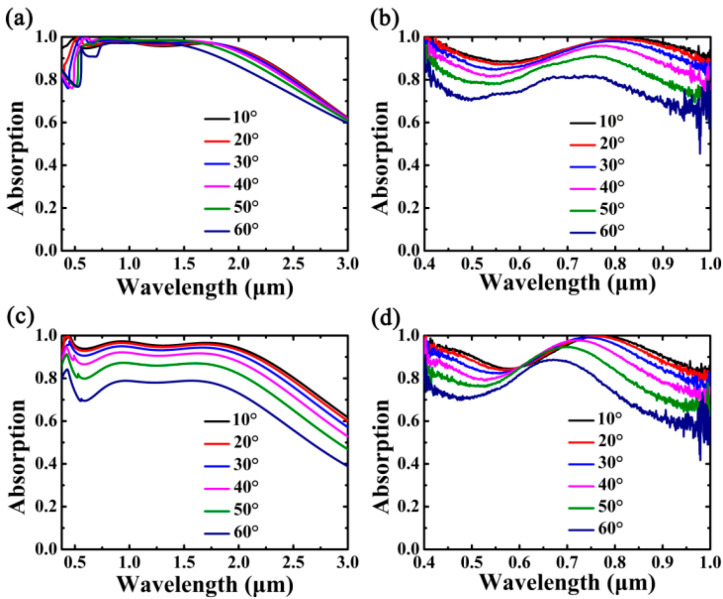
Absorption curves of the Cr-based MA at oblique incidence. Other parameters are kept the same as Figure 1a. (**a**,**b**) are the simulated and measured absorption of TM polarization, respectively. (**c**,**d**) are the simulated and measured absorption of TE polarization, respectively.

We further characterized the incident angle dependence of the Cr-based MA under the illumination for both the TM and TE polarizations. As shown in Figure 5a, the absorption spectra exhibit omnidirectional features in the designed wavelength region in the case of TM polarization. The absorbing properties can be kept almost the same even the incident angle *θ* is significantly altered, and an average absorption of 90.8% can be obtained in the wavelength range of 380.0–2137.5 nm as *θ* = 60°. In Figure 5b, it can be seen that the Cr-based MA shows good absorption performances in experiment within the range of 400–1000 nm. In Figure 4c, it can be seen that the absorption performances of the Cr-based MA can be maintained wellfor TE polarization within ±60°; however, the intensity of the absorption and the absorption bandwidth drops quickly whenthe incident angle is larger than 60°. In the case of TE wave illumination, the tangential component of magnetic incident field decreases as *θ* > 60°, the magnetic coupling and the incident magnetic flux between the metallic films is reduced, thus less light energy could be absorbed effectively due to the impedance mismatch [61,62,63]. As can be seen in Figure 5d, the measured curves show broadband absorption features. although they vary more obviously as *θ* increases, in comparison with those of the TM polarization.

**Figure 6:**
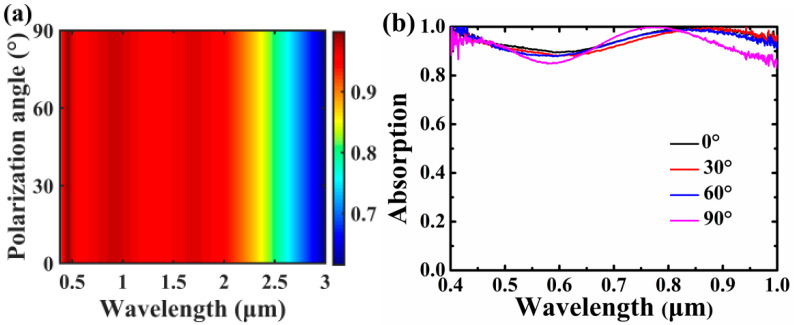
Absorption spectra of the Cr-based MA under the variation of polarization angle. Other parameters are kept the same as Figure 1a. (**a**) Theoretical absorption 2D map as a function of polarization angle. (**b**) Measured absorption spectra as a function of polarization angle.

The influences of polarization angle on absorption performances of the Cr-based MA are shown in Figure 6. As can be seen in Figure 6a, absorption of the Cr-based MA is immune to the variation of the polarization angle due to the symmetrical topology. As the polarization angle is altered from 0° to 90°, that is, from TM polarization to TE polarization, ultrabroadband absorption performance of the Cr-based MA remains the same. In Figure 6b, it can be seen that the measured absorption spectra are insensitive to the variation of the polarization angle. Therefore, the proposed Cr-based MA is angle-and polarization-insensitive and can be operated well for both the TM and TE polarizations.

## 5. Summary

In conclusion, we have designed, fabricated, and characterized an ultrabroadband Cr-based MA in the visible to near-infrared region.Ultrabroadband absorption with RAB = 139.6% and average absorption of 96.4% can be realized within the range of 380.0–2137.5 nm in design, and an average absorption of 93.7% can be achieved in the 400–1000 nm wavelength range in experiment. The absorption enhancement results from the co-excitations of LSP and PSP and their synergistic absorption effects, which ensure the ultrabroadband features via the Cr-based MA with subwavelength thickness. The Cr-based MA is very robust to variations of the geometrical parameters, and ultrabroadband performances can be maintained even if the structural parameters are significantly altered. In addition, our device exhibits omnidirectional features in the designed region within ±60° for the TM polarization, and angle-and polarization-insensitive absorption can be operated well under both TM and TE-polarized light illumination. The proposed scheme combines the advantages of both LSP and PSP modes in a low-cost andcomparatively simple architecture, which will be beneficial for various interesting applications such as photodetection, energy harvesting, and thermalemitters.
